# Molecular dynamics simulation analysis of a modelled spermidine synthase from *Yersinia pseudotuberculosis* docked with cyclohexylamine

**DOI:** 10.6026/973206300210210

**Published:** 2025-02-28

**Authors:** Krishna Kuna, Srinivas Ganta, Pavan C. Akkiraju, Sudheer Kumar Dokuparthi, Sardar Hussain, Sreenivas Enaganti

**Affiliations:** 1 Department of Chemistry, University College of Science, Saifabad, Osmania University, Hyderabad-500004, Telangana, India; 2 Department of RNA Therapeutic, SciGen Pharmaceutical Inc., Hauppauge, NewYork-11788, United States of America; 3 Department of Biotechnology, School of Allied Healthcare Sciences, Malla Reddy University, Hyderabad-500043, Telangana State, India; 4 Biogenic Products Private Limited, Hyderabad-500007, Telangana State, India; 5 Department of Biotechnology, Maharani's Science College for Women, Mysuru 570005, Karnataka, India; 6 Department of Bioinformatics Averinbiotech Laboratories, No 208, 2nd Floor, Windsor Plaza, Nallakunta, Hyderabad-500044, Telangana, India

**Keywords:** Aminopropyl transferase spermidine synthase, spermidine synthase, *Yersinia pseudotuberculosis*, homology modeling, molecular docking, molecular dynamics

## Abstract

The gram-negative bacterium *Yersinia pestis* is the causative agent of plague 1 and has been responsible for major pandemics in the past. Therefore, it is of interest to document the molecular docking and simulation analysis of spermidine synthase from *Yersinia* pseudotuberculosis with cyclohexylamine. The sequence and structure analysis showed an abundance of Leu, Val, Gly, Glu and Ala, the least presence of Trp and Cys, higher negatively charged residues and a GRAVY score of -0.125, suggesting the stability of the protein. The cyclohexylamine conformer 4-fluorocyclohexan-1-amine (CID 21027526) showed optimal binding features (-4.7 kcal/mol). Moreover, molecular dynamics simulation confirmed the stability of the ligand binding pocket for further validation and consideration in drug design and development.

## Background:

The plague pandemic in the last century has created an alarming situation for human health. Such a pandemic proved the importance of our preparedness and the requirement of our enhanced scientific understanding in the management of health crises [1- 2]. The gram-negative bacterium *Yersinia pestis* is the causative agent of plague 1 and has been responsible for major pandemics in the past [3]. *Yersinia pseudotuberculosis* causes Far East scarlet-like fever in humans and GI tract infections, specifically in children [4-5]. This bacterium also infects animals. The amino prenyl transferase spermidine synthase (SpdS) is considered a promising drug target for pathogens, including *Yersinia*. The molecular and biochemical characterization of this enzyme was done in many pathogens, such as the malarial parasite *Plasmodium falciparum* [6] and *Leishmania* [7- 8]. Polyamine metabolism is vital for these pathogens and a crucial factor for their virulence [8]. Therefore, inhibiting this crucial enzyme through precise molecular targeting can help to prevent the pathogens' survival. This enzyme is present immediately downstream of Ornithine decarboxylase (ODC), a pyridoxal phosphate-dependent enzyme that acts as the rate-limiting enzyme for polyamine synthesis in the polyamine pathway [9]. The enzyme transfers the aminopropyl group of decarboxylated S-adenosylmethionine (dcAdoMet) to putrescine or to spermidine forming spermidine or spermine, respectively. Hence, functionally, spermidine synthase is a crucial enzyme (EC 2.5.1.16) that catalyzes the transfer of the propyl amine group from S-adenosyl methionine amine to putrescine in the biosynthesis of spermidine. The systematic name is S-adenosyl 3-(methyl thio) propylamine: putrescine 3-aminopropyltransferase and it belongs to the group of aminopropyl transferases. Generally, it does not need any cofactors. Most spermidine synthases exist in solution as dimers. However, structural and mechanistic variations of the enzymes are observed in different species or organisms. Earlier studies have considered targeting and analysing various strains of *Yersinia* for potential drug-target proteins, experimenting with various small molecules as inhibitors and probable ways to counter the infection spread [10 - 11]. Therefore, it is of interest to document the molecular docking and simulation analysis of spermidine synthase from *Yersinia pseudotuberculosis* with cyclohexylamine derivatives.

## Methodology:

The structural and functional characterization of the target protein Spermidine Synthase (SPDS) in *Yersinia pseudotuberculosis* is of interest. We validated the target protein as a potential drug target candidate against *Yersinia*.

## Protein sequence identification and collection:

The sequences were searched with specific search terms using boolean operators. The protein sequences were collected from the National Center for Biotechnology Information (NCBI) protein database [12]. All sequences were collected in FASTA format for further analysis.

## Sequence characterization and target identification:

All collected sequences were subjected to sequence-based biochemical and biophysical characterization including the amino acid composition, theoretical estimation of molecular weight, theoretical pI, total number of negatively charged residues (Asp + Glu), total number of positively charged residues (Arg + Lys), extinction coefficients( at 280 nm measured in water), Abs 0.1% (=1 g/l) (assuming all pairs of Cys residues form cystines), estimated half-life (mammalian reticulocytes, in vitro), instability index, instability index classification, aliphatic index and grand average of hydropathicity (GRAVY) [13]. A phylogenetic tree was developed for the collected protein sequences using the neighbor-joining method available in MEGA 11 tool to understand their similarities and diversities [14]. The target sequence was identified (Accession: Q66EH3.1).

## Protein structure modeling:

## Template search and selection:

To construct the three-dimensional structure, a template-based homology modeling approach was adopted [15- 16]. Template searching was done based on the sequence identity and similarity and based on the position-specific scoring matrix (PSSM) or profile [17] to attain higher accuracy. PSI-BLAST (Position-Specific Iterative Basic Local Alignment Search Tool) [18] was used to identify the closest template protein structure available in the protein data bank (PDB) [19]. Mode base was also considered for a plausible template search [20].

## Protein structural model development:

The protein modeling was done using spatial restraint-based homology modeling in the Modeller 10.4 environment [21- 22]. All the initially selected templates were compared based on the sequence structural identity and quality. The crystal structure of the spermidine synthase from *E. coli* (PDB ID: 3O4F, Chain A) was selected as the template for structure development. The structure had a 2.90 Å resolution with 0.241 R-value free [23]. The model was developed using target-template alignment generation, followed by spatial restraint-based 3D coordinate assignment to the target sequence and structural quality evaluation using the Modeller objective function [24], discrete optimized protein energy (DOPE) [25] and GA341 was scoring method [26]. A total of 10 structures were generated and the best model was selected based on the mentioned scores.

## Structural quality evaluation and loop refinement:

The final selected structure was inspected for structural quality and proper chirality of the atoms using the Ramachandran plot in the Procheck server [27-28]. Based on the Ramachandran plot analysis and the increased DOPE score, potential structural regions requiring structural refinement were identified and loop modeling was done for the final structure through simultaneous loop refinement and energy minimization. The selected best model after loop refinement was subjected to structural quality assessment and was used in this study.

## Pocket analysis:

The developed protein structure was analyzed in detail for sequential and structural features. The computed atlas of the protein surface topographical analysis was done using a probe radius of 1.4 Å [29]. Several pockets in the protein were identified and the largest pocket was studied in detail.

## Docking analysis:

## Ligand collection and molecular mechanical calculations:

The available literature related to spermidine synthase inhibition in parasites [30] and plants [31] suggested that cyclohexylamine, N-(3-aminopropyl)cyclohexylamine (APCHA) and trans-4-methyl cyclohexylamine are the major inhibitors that are regularly used to inhibit the function of spermidine synthase. We have focused on the cyclohexylamine group of inhibitors. Altogether, 129 compounds were collected in .sdf format (2D and 3D coordinates) from the PubChem structural database [32]. All the molecular structures of the compounds were checked for structural quality and molecular mechanical calculations were done using the MMFF94 force field with the steepest descend algorithm for 500 iterations in Avogadro software [33].

## Protein structure preparation and molecular docking:

Hydrogen atoms were added to the protein structure, followed by the addition of Kollman charges and Gasteiger charges and minimization using the AutoDock Tools [34]. The docking pocket was decided after the AutoDock grid setting considering the molecular pocket analysis outcomes. The AutoDock Vina was used for docking the molecules to the protein pocket [34] and VMD was used for protein visualization and analysis [35].

## MD Simulation:

This study involved a molecular dynamics (MD) simulation for 100 ns of the ligand-protein complex to assess the binding efficacy of the compound to the target protein. We implemented the CHARMM36 force field [36] in the GROMACS 2022.4 package [37] for conducting MD simulations on the complex. The Cgenff server [38] was employed to produce the topologies and parameter files for the ligand. The Particle Mesh Ewald (PME) approach [39] was utilized for the calculation of electrostatic forces. Solvation of ligand-protein combination was performed using the transferable intermolecular potential with a three-point (TIP3P) water model. The complex was recreated using a dodecahedron-shaped box, with a buffer distance of 1Å [40]. The neutrality of the system was achieved through the incorporation of Na+ and Cl- ions into the system. By energy reduction through 5,000 iterations of the steepest descent method, the presence of unfavorable connections and collisions within the protein structure was mitigated. The LINCS method [41] was employed to completely remove all hydrogen bonds, after which the entire system was subjected to heating at a temperature of 310 K. Following the minimization of energy, the complex underwent two successive equilibration stages, a 1 ns equilibration under the NVT ensemble and a 1 ns equilibration under the NPT ensemble. The velocity-rescaling approach [42] was employed to incorporate temperature coupling. The Parrinello-Rahman pressure method [43] was employed to maintain constant pressure. During the 100 ns production run, a system that had reached equilibrium was used. Analysis of the complete system was done using GROMACS analytical modules, focusing on structural and conformational aspects. Post-MD analysis included RMSD, RMSF, SASA and hydrogen bonds.

## Results:

## Target protein selection:

The target protein for this study was selected after a thorough literature search and analysis of the public domain databases such as the NCBI protein database and the Swissprot database. The specific search term used was "amino propyl transferase spermidine synthase (SpdS) AND *Yersinia pestis*" and "amino propyl transferase spermidine synthase (SpdS) AND *Yersinia*". The search yielded 6 protein sequences bearing accession numbers Q8ZBJ8.1, A9R1H4.1, Q1CLX1.1, Q1C3U7.1, A4TPU4.1 and Q66EH3.1. 

## Protein physicochemical properties:

All the proteins were subjected to physicochemical analysis to understand their biochemical and biophysical similarities and differences. All these protein sequences had a higher percentage of Leu, Ala, Gly and Val (Figure 1 - see PDF) and a lower percentage of Trp, Cys and Met (Figure 1 - see PDF). All 6 proteins were of similar length having 296 amino acids each with slight variation in the molecular weight (Table 1). The theoretical PI was 4.87 as estimated from the sequences. The ratio of the total number of negatively charged residues was more than the positively charged residues for all these proteins (Table 1).The calculation half-life was approximately 30 hours and the protein was found stable with an instability index of 39.69. Computed higher aliphatic index (>80) suggested that the protein is thermostable. The estimated GRAVY scores suggested that the SPDS proteins are hydrophilic and the proteins are likely to be of globular type (Table 1). The collected 6 sequences had almost no difference at the sequence level; hence, a phylogenetic analysis using the UPGMA method revealed that there is a slight distance present with sequence accession number Q66EH3.1 and all other 5 sequences (Figure 2 - see PDF). The sequence bearing accession number Q66EH3.1 was selected as the target protein. This 296 amino acid-containing protein sequence was from *Yersinia pseudotuberculosis*.

## Protein structure development:

## Template search:

The template was searched in Modbase and PDB database. A position-specific scoring matrix-based position-specific interactive search was conducted for 5 iterations and the closest protein having a crystallographic structure with the lowest E-value was selected as the template (Table 2).

## Template evaluation:

The top 5 templates were selected and compared using the weighted pair-group average clustering method and sequence comparison analysis. This analysis included the sequential and structural differences among the templates along with their resolution, R factors and other sequential and structural features. The "A" chain of the crystal structure of spermidine synthase (PDB ID: 3O4F) from *E. coli* with a resolution of 2.9 Å was selected as the template to build the model.

## Model development:

The model was developed after a precise target-template alignment (Figure 3 - see PDF). The conserved segments are presented with (*). The sequence identity was 81.5%. A total of 10 models were developed and all the models were evaluated using an objective function, Discrete Optimized Protein Energy (DOPE) score and GA341 score (Table 3). The 7^th^ model was found to be the best model out of the 10 generated models. The structural model was selected and validation was done. The DOPE profile of the model is shown in Figure 4 (see PDF).

## Structural model validation:

The initial model was selected based on the low objective function, DOPE score and GA341 values of 1.0 (Table 3). Later the structure was validated with Ramachandran plot and other methods. Further analysis revealed that the requirement of loop refinement at residue positions: 9-16, 146-151 and 162-170.

## Structural loop refinement:

A total of 10 different structures were developed while optimization was done simultaneously for the 3 loop segments (Table 4). The best model was selected based on the low DOPE score and further analyzed for structural components visually using VMD 1.9 [35] (Figure 5A - (see PDF) and Figure 5C - (see PDF)) and structural quality evaluation was done using the Ramachandran plot (Figure 5B - (see PDF)). The target-template alignment is shown here where orange is the template and the cyan color is for the final loop refined structural model (Figure 5D - see PDF).

## Structural evaluation:

The structure was evaluated using the Ramachandran plot and other methods. The overall structural quality suggested that out of 296 amino acids of the target protein, 92.7% amino acids were in the core region of the Ramachandran plot, 5.4% in the allowed region, 0.8% generously allowed region and 1.1% amino acids were in the disallowed region (Figure 5B - see PDF). The template and target protein sequence and structure residue-specific RMSD were mostly distributed between 1Å except for a few residues (Figure 6A - see PDF), thus suggesting the close sequential and structural similarity of the template and target proteins. The local structural difference between the template and the target protein was done using the Q per residue (Qres) calculations (Figure 6B - see PDF). Sequence conservation and the percent identity are shown for the template and the target protein structures (Figure 6C - (see PDF) and Figure 6D - (see PDF)).

## Structure analysis:

Spermidine synthase (EC number: 2.5.1.16) is an all-beta domain protein with additional tetramerization at the N-terminal. This protein catalyzes the biosynthesis of spermidine from arginine and methionine at the last step of the biosynthesis process. The spermidine aminopropyl transferase (EC number: 2.5.1.22) catalyzes the production of spermine from the spermidine through the cofactor decarboxylated S-adenosylmethionine. The secondary structure analysis of the developed protein showed an almost equivalent share of the beta-strand (27.7%) and alpha helix (27.4%) with 82 and 81 amino acids out of 296 total amino acids, respectively. The structural formation of the 3-10 helixes was done by only 3.4% (10) of the total amino acids. Other structural formations including loops were developed by 41.6% of the total amino acids of the protein. The structure had 3 beta sheets with 2, 4 and 7 strands, respectively (Figure 5C - see PDF). Two beta-sheets were antiparallel and one was mixed type. The formation of 3 beta-alpha-beta motifs was observed in the structure. Five beta hairpins were observed at Glu5-Tyr8, Asn23-Glu28, Leu35-Asn40, Gly44-Leu49, Asp215-Ala222 (Strand 1) and Tyr18-Val21, Leu35-Asn40, Gly44-Leu49, Val52-Thr56, Ile230-Thr237 (Strand 2). Also, five beta bulges, 13 strands, 12 helices along with 10 helix-helix interactions and 30 beta turns were observed in the modeled structure. A total of 3 inverse gamma turns were observed between Thr30-His32, His32-Asp34 and Ala257-Leu259.

## Pocket analysis of the protein:

A total of 53 pockets were identified in the protein structure. The largest pocket was (Figure 7A - see PDF) 631.69 surface area volume. The amino acids that were part of the pocket are shown in Figure 7A (see PDF). The other major pockets are shown in Figure 7B (see PDF).

## Docking analysis:

The present study focused on the cyclohexylamine series of ligands. However, docking was done with the N-(3-aminopropyl) cyclohexylamine (APCHA) and trans-4-methyl cyclohexylamine also. A total of 142 ligands were considered out of which 3 were Cyclohexylamine, N-(3-aminopropyl) cyclohexylamine (APCHA) and trans-4-methyl cyclohexylamine. A total of 80 conformers of Cyclohexylamine were considered and the rest of the ligands were similar compounds to cyclohexylamine (Supplementary 1).

A total of 9 docking poses were generated for each case using an exhaustiveness value of 8 during docking exercises (Figure 8 - see PDF). The analyses of the docking results suggested that the binding affinity scores were mostly between -3.83 and -3.74 or -3.74 and -3.64. However, the highest binding affinity score ranged from -4.5 to -4.43. Most of the cyclohexylamine conformers showed better docking affinity scores with values of ~ -4.0. However, compounds other than cyclohexylamine conformers such as 4-fluorocyclohexan-1-amine (CID- 21027526) also showed greater affinity towards the protein binding. The specific interactions of this compound and the cyclohexylamine conformer are shown in Figure 9(see PDF). The observed bond types were either alkyl type or van-der Waals bond formations.

## Protein-ligand complex MD Simulation:

## RMSD of Protein:

The RMSD plot (Figure 10A - see PDF) of the protein-ligand complex exhibits an early increase in deviation over the first 20 nanoseconds (ns), signifying substantial conformational alterations as the protein accommodates the ligand binding. After this interval, the system attains a stable condition, with the RMSD oscillating around 0.6 nm for the duration of the simulation, which lasts up to 100 ns. The fluctuations indicate that although the protein-ligand combination had reached equilibrium, the protein underwent slight structural modifications due to natural thermal fluctuations. The complex stabilizes after 20 ns (Figure 10A - see PDF, exhibiting no significant structural variations thereafter.

## RMSD of Ligand:

The RMSD plot (Figure 10B - see PDF) for the ligand indicates substantial conformational alterations during the simulation. Initially, the RMSD was increased during the first 20 nanoseconds (ns) and attained ~10 nm, signifying substantial displacement from its original position. Throughout the experiment, the RMSD consistently increased, exhibiting significant position changes up to 40 nm at ~70 ns. After this juncture, the ligand seems to attain stability, oscillating around 40 nm for the duration of the simulation, extending to 100 ns. The elevated RMSD values imply that the ligand experiences significant structural alterations, potentially signifying flexibility. As the simulation progressed beyond 20 ns, the RMSD exhibited a gradual increase, signifying additional large-scale movements. Between 20 ns and 70 ns, the RMSD increased steadily, ultimately attained ~40 nm. This significant increase indicates that the ligand is undergoing considerable structural flexibility or major displacements from its original binding location. This behavior may suggest that the ligand is weakly associated or is navigating various binding modes within the pocket, maybe transitioning between distinct binding conformations. The RMSD stays consistently low, oscillating at ≤1 nm. This suggests that the ligand remains comparatively stable throughout this duration, exhibiting minimal structural change from its original structure.

## RMSF of the Protein:

The Root Mean Square Fluctuation (RMSF) calculation evaluates the flexibility of individual residues in the protein throughout the 100 ns molecular dynamics (MD) simulation (Figure 10E - see PDF). The RMSF values quantify the mean positional variations of each residue over the simulation period. A significant number of protein regions exhibited low RMSF values, generally under 0.5 nm. The areas encompassing residues 0 to 50, 100 to 200 and 250 to 290 exhibited relative stability, signifying that these segments of the protein preserve a consistent shape throughout the simulation. Residues with elevated RMSF values are typically situated in loops, turns, or termini, where the protein structure is less restricted, facilitating increased mobility. The RMSF plot (Figure 10E - see PDF) demonstrates that the majority of protein residues displayed minimal flexibility, indicating that the protein's overall structure remains stable throughout the 100 ns simulation. However, slight variations in RMSF were observed for the flexible loops or termini regions, which was obvious due to increased mobility in molecular dynamics simulations because of diminished structural restrictions.

## SASA of the Protein:

Solvent Accessible Surface Area (SASA) mapping was done for 100 ns molecular dynamics (MD) simulation (Figure 10F - see PDF). During the initial 20 ns of the simulation, the SASA rises from ~150 nm^2^ to ~170 nm^2^. This increase signifies that the protein's solvent-exposed surface area expanded, implying potential unfolding or reorganization of surface residues, permitting greater exposure of the protein to the solvent. Later, the SASA oscillated between 160 nm^2^ and 165 nm^2^, exhibiting minor peaks and troughs that signified the inherent dynamic behavior of the protein. The variations suggested minor conformational alterations of the protein. However, later, the solvent-exposed regions stabilized, indicating that the protein attained an equilibrated structure after ~20 ns. The SASA displayed slight fluctuations during the simulation, perhaps owing to localized structural modifications or the mobility of flexible regions such as loops or termini that either reveal or conceal specific areas of the protein.

## Discussion:

Spermidine synthase is a crucial enzyme that acts in the last biosynthesis step of spermidine, a polyamine involved in multiple cellular functions. The role of spermidine includes the stabilization of DNA and RNA, modulating the process of autophagy and eukaryotic initiation factor 5A formation [44]. Earlier studies on human spermidine synthase have shown that the enzyme exists in a dimer form containing two identical subunits with a large pocket [45]. The modelled structure also showed a large pocket with a higher pocket volume (631.69 Å3) and surface area (618.37 Å2). Most of the interacting amino acids with ligands were observed towards the N-terminal of the protein. More beta turns were observed towards the N-terminal of the protein after the 200th residue. The crystal structure of spermidine synthase has been studied from different sources with their respective substrate binding capacities [45-46]. The amino-propyl transferase family contains many spermidine synthases with variations in structures and mechanistic differences as observed in different organisms [47]. The structure of spermidine synthase from different organism sources has notable structural and functional differences. Therefore, spermidine synthase from bacterial sources such as *Yersinia* may have different structural features and functional mechanisms. Hence, to target *Yersinia* by inhibiting spermidine synthase, we have developed the protein structure using homology modelling and studied the structure in detail. Also, we investigated possible ways of inhibition with the well-known inhibitors that are likely to interact or inhibit spermidine synthases. This is the first theoretical model development of spermidine synthase from *Yersinia pseudotuberculosis*. We have observed that the modelled structure was highly similar and relevant to the *E. coli* template structure (>81% identity) as both belong to the bacterial origin. We have extensively examined the binding of cyclohexylamine conformers (n=81) and other similar compounds (n=48) (Supplementary 1), to understand the binding mechanism of the molecules in the protein pocket. The residues towards the N-terminal were found to interact with the ligands. Evaluation of the structural features showed similarity to other reported structures, such as the presence of putrescine amino-propyl-transferase (PAPT) fold structural features and a Rossmann-like fold [46]. We have observed that several residues such as Ala296, Glu9, Leu11, Tyr227, Val52 and His12 are repetitively interacting with the cyclohexylamine and other compounds considered as ligands in this study. These results suggest that such residues present in the large pocket are highly interactive and can have easy access to the ligand molecules for binding and various bond formations. MD simulation (100 ns) of the best-docked compound (CID 21027526) suggested that the ligand was docked properly and had multiple conformational changes over the simulation period, perhaps due to the small ligand size and availability of space in the pocket. However, the binding was observed stable suggesting the protein-ligand interaction to be thermodynamically favorable. Earlier experimental studies in mammalian and rat tissues considered cyclohexylamine inhibitors as potential inhibitors for spermidine synthase [48-49]. Our results also predicted the possible potential inhibition capacity of the inhibitor for the modeled protein from *Yersinia*.

## Conclusion:

The cyclohexylamine conformer 4-fluorocyclohexan-1-amine (CID 21027526) showed optimal binding features (-4.7 kcal/mol) with stability with the spermidine synthase from *Yersinia pseudotuberculosis* for further validation and consideration in drug discovery.

## Figures and Tables

**Table 1:** Computed physicochemical properties of the collected Yersinia protein sequences

**Properties**	**Q8ZBJ8.1**	**A9R1H4.1**	**Q1CLX1.1**	**Q1C3U7.1**	**A4TPU4.1**	**Q66EH3.1**
Number of amino acids	296	296	296	296	296	296
Molecular weight	33166.45	33166.45	33166.45	33166.45	33166.45	33182.45
Theoretical pI	4.87	4.87	4.87	4.87	4.87	4.87
Total number of negatively charged residues (Asp + Glu)	37	37	37	37	37	37
Total number of positively charged residues (Arg + Lys)	20	20	20	20	20	20
Extinction coefficients( at 280 nm measured in water)	30620	30620	30620	30620	30620	32110
Abs 0.1% (=1 g/l)(assuming all pairs of Cys residues form cystines)	0.923	0.923	0.923	0.923	0.923	0.968
Estimated half-life(mammalian reticulocytes, in vitro)	30 hours	30 hours	30 hours	30 hours	30 hours	30 hours
Instability index	39.69	39.69	39.69	39.69	39.69	39.12
Instability index Classification	Stable	Stable	Stable	Stable	Stable	Stable
Aliphatic index	86.62	86.62	86.62	86.62	86.62	86.62
Grand average of hydropathicity (GRAVY)	-0.111	-0.111	-0.111	-0.111	-0.111	-0.125

**Table 2:** Template search results for the target sequence (ID: Q66EH3.1) after the 5th iteration of the PSI search

**Query protein**	**Template PDB ID**	**Percentage identity of sequence**	**Template length**
Q66EH3.1	3O4F_A	80.556	288
Q66EH3.1	3RW9_A	34.146	287
Q66EH3.1	2O05_A	34.146	287
Q66EH3.1	6O65_A	35.889	287
Q66EH3.1	6O63_A	35.889	287
Q66EH3.1	6O64_A	35.294	289
Q66EH3.1	1XJ5_A	35.192	287
Q66EH3.1	8IYI_A	32.281	285
Q66EH3.1	2B2C_A	37.363	273
Q66EH3.1	4YUV_A	33.448	290

**Table 3:** Structural model development based on the satisfaction of the spatial restraints

**Model Number**	**Objective Function**	**Template Sequence Identity (%)**	**DOPE**	**GA341**
Q66EH3_1.B99990001	1861.0173	81.56	-33987.1	1
Q66EH3_1.B99990002	1652.5259	81.56	-33997.5	1
Q66EH3_1.B99990003	1816.4247	81.56	-33945.4	1
Q66EH3_1.B99990004	1694.8091	81.56	-33782.9	1
Q66EH3_1.B99990005	1694.8701	81.56	-33969.1	1
Q66EH3_1.B99990006	1738.1781	81.56	-33823.7	1
Q66EH3_1.B99990007	1628.3859	81.56	-34162.6	1
Q66EH3_1.B99990008	1644.6112	81.56	-33913.9	1
Q66EH3_1.B99990009	2180.6584	81.56	-33502.8	1
Q66EH3_1.B999900010	1646.162	81.56	-33765.1	1

**Table 4:** Loop refinement results with objective function and DOPE scores

**Model Number**	**Objective Function**	**DOPE scores**
Q66EH3_1.BL00010001	1320.4248	-33515.2
Q66EH3_1.BL00020001	128.7557	-33997.1
Q66EH3_1.BL00030001	152.2388	-34198.3
Q66EH3_1.BL00040001	3889.2461	-32788.9
Q66EH3_1.BL00050001	2683.6182	-33057.9
Q66EH3_1.BL00060001	551.7504	-33732.8
Q66EH3_1.BL00070001	1180.9202	-33385
Q66EH3_1.BL00080001	877.293	-33751.2
Q66EH3_1.BL00090001	112.8272	-34152.7
Q66EH3_1.BL00100001	2031.5222	-33583.8
